# New opportunities in tuberculosis prevention: implications for people living with HIV

**DOI:** 10.1002/jia2.25438

**Published:** 2020-01-08

**Authors:** Lucia González Fernández, Esther C Casas, Satvinder Singh, Gavin J Churchyard, Grania Brigden, Eduardo Gotuzzo, Wim Vandevelde, Suvanand Sahu, Sevim Ahmedov, Adeeba Kamarulzaman, Alfredo Ponce‐de‐León, Beatriz Grinsztejn, Susan Swindells

**Affiliations:** ^1^ HIV Co‐Infections and Co‐Morbidities The International AIDS Society Geneva Switzerland; ^2^ Southern Africa Medical Unit Médecins Sans Frontières Cape Town South Africa; ^3^ HIV Department World Health Organization Geneva Switzerland; ^4^ Aurum Institute Parktown South Africa; ^5^ School of Public Health University of Witwatersrand Johannesburg South Africa; ^6^ Advancing Care and Treatment for TB/HIV South African Medical Research Council Parktown South Africa; ^7^ Department of Tuberculosis International Union Against Tuberculosis and Lung Disease Geneva Switzerland; ^8^ Department of Medicine and Director of the “Alexander von Humboldt” Institute of Tropical Medicine and Infectious Diseases Peruvian University Cayetano Heredia Lima Peru; ^9^ Global Network of People living with HIV (GNP+) Cape Town South Africa; ^10^ Stop TB Partnership Geneva Switzerland; ^11^ Bureau for Global Health, Infectious Diseases, TB Division USAID Washington DC USA; ^12^ Department of Medicine University of Malaya Kuala Lumpur Malaysia; ^13^ Infectious Diseases Department Instituto Nacional de Ciencias Médicas y Nutrición Salvador Zubirán Mexico City Mexico; ^14^ Instituto Nacional de Infectologia Evandro Chagas Fiocruz Brazil; ^15^ University of Nebraska Medical Center Omaha NE USA

**Keywords:** TB, HV care continuum, differentiated care, public health, co‐infection, treatment

## Abstract

**Introduction:**

Tuberculosis (TB) is a leading cause of mortality among people living with HIV (PLHIV). An invigorated global END TB Strategy seeks to increase efforts in scaling up TB preventive therapy (TPT) as a central intervention for HIV programmes in an effort to contribute to a 90% reduction in TB incidence and 95% reduction in mortality by 2035. TPT in PLHIV should be part of a comprehensive approach to reduce TB transmission, illness and death that also includes TB active case‐finding and prompt, effective and timely initiation of anti‐TB therapy among PLHIV. However, the use and implementation of preventive strategies has remained deplorably inadequate and today TB prevention among PLHIV has become an urgent priority globally.

**Discussion:**

We present a summary of the current and novel TPT regimens, including current evidence of use with antiretroviral regimens (ART). We review challenges and opportunities to scale‐up TB prevention within HIV programmes, including the use of differentiated care approaches and demand creation for effective TB/HIV services delivery. TB preventive vaccines and diagnostics, including optimal algorithms, while important topics, are outside of the focus of this commentary.

**Conclusions:**

A number of new tools and strategies to make TPT a standard of care in HIV programmes have become available. The new TPT regimens are safe and effective and can be used with current ART, with attention being paid to potential drug‐drug interactions between rifamycins and some classes of antiretrovirals. More research and development is needed to optimize TPT for small children, pregnant women and drug‐resistant TB (DR‐TB). Effective programmatic scale‐up can be supported through context‐adapted demand creation strategies and the inclusion of TPT in client‐centred services, such as differentiated service delivery (DSD) models. Robust collaboration between the HIV and TB programmes represents a unique opportunity to ensure that TB, a preventable and curable condition, is no longer the number one cause of death in PLHIV.

## 1 Introduction

Tuberculosis (TB) is a leading cause of mortality among people living with HIV (PLHIV). This population contributes substantially to the global TB burden, with a higher risk of progressing from TB infection to disease than HIV‐negative people 1, 2, 3. In 2015, WHO launched the “End TB” strategy, setting ambitious targets of a 90% reduction in TB incidence and 95% reduction in mortality by 2035. These aspirations were included in the Sustainable Development Goals 4, 5. In 2018, the UN High‐Level Meeting (UNHLM) on Tuberculosis galvanized the global efforts to prevent TB by setting renewed targets to provide TB preventive therapy (TPT) to 30 million people by 2020 including six million PLHIV 6, 7. Following the UNHLM, a Lancet Commission on TB: “Building a tuberculosis‐free world” underscored the rollout of TPT among PLHIV as a cornerstone for achieving success in the global TB control efforts, complemented with active TB case‐finding and initiation of prompt and effective TB treatment 4, 8. Evidence shows that TPT reduces TB incidence, and also mortality in PLHIV up to 37% independent of ART and is a cost‐effective intervention, even using shorter regimens 9, 10, 11. Moreover modelling studies underpin expanded use of TPT as an essential intervention to reduce the global TB burden, with great effect in high HIV prevalence settings 12, 13, 14. Despite these facts, TPT scale‐up within HIV programmes has been appallingly low and insufficient to effectively decrease the TB burden in PLHIV 8, 10, 15, 16, 17, 18, 19, 20. With an estimated 920,000 new TB infections among PLHIV and 300,000 deaths (a third of HIV‐related deaths globally in 2017), the UN target to reduce TB deaths among PLHIV by 75% by 2020 will remain unmet 21. Barriers preventing TPT rollout are related to access to patient‐friendly formulations, including for children, difficulties of ruling out active TB, unfounded fear of development of drug resistance, perceived provider beliefs that drug toxicity outweighs the benefit, or competing priorities to deliver antiretroviral treatment (ART) 22, 23.

We present a summary of the current TPT options and innovations in this field. We discuss challenges and opportunities to scale‐up TPT within HIV programmes. Finally, we call for action to stakeholders in HIV high‐burden settings to improve access to new TPT, exploring innovative ways to deliver TPT using differentiated services and demand creation approaches.

## 2 Discussion

### 2.1 Research and innovation in TB prevention: implications for PLHIV

The understanding of the dynamics of TB infection and activation keeps improving and has evolved to describe a continuum between “latent” and “active” states that cover different scenarios between latent subclinical infection to clinically active disease 3, 24, 25, 26. While much remains to be done in the quest for diagnostic technologies to provide a public health solution that can effectively find cases of TB infection at high risk of progression, the field of treatment has advanced significantly 26. Antiretroviral therapy alone prevents TB at individual and programmatic levels, and is now recommended for all people with HIV infection, including during additional preventive therapy for TB 23, 27, 28.

TPT in PLHIV aims to prevent progression to clinical disease, reducing TB‐related mortality, morbidity, including hospitalizations and ultimately decrease TB transmission 11, 17, 18, 29, 30, 31, 32. In this context, TPT should be integrated as a standard package of care including routine TB screening, early diagnosis and treatment of TB disease, effective ART, and education and access to harm reductions programmes such as use and abuse of alcohol and drugs, including tobacco 26, 33.

Much progress has been made to make new, shorter and better tolerated TPT regimens available by including rifapentine 27, 34, 35. A 3‐month course of weekly rifapentine and isoniazid (3HP) has proved as effective as nine months of isoniazid (INH), was better tolerated, had higher completion rates and is safe and effective in PLHIV 27, 34. This regimen can be co‐administered with dolutegravir (DTG) without dose adjustment and it is included in the 2018 WHO recommendations for the treatment of latent TB infection 27, 36. The most recent, and not yet included in international recommendations, uses 1‐month daily rifapentine plus isoniazid (1HP) and has shown non‐inferiority to nine months of isoniazid alone for preventing TB in HIV co‐infected patients, with lower incidence of adverse events and better adherence to treatment completion 35.

However today, the most commonly used TPT regimen contains classically one drug, isoniazid, administered for six to twelve months and can be co‐administered with any ART regimen. The INH‐CoTrim‐VitB6 combination may offer benefits with respect to pill burden 32, 37. The length of the course and repetition of courses remain as important questions, for which current evidence goes as far as 36 months as a proxy for life‐long treatment 27, 38. Current WHO recommendations support the use of isoniazid preventive treatment (IPT) for six months, while thirty‐six months remains a recommendation for high incidence areas 38. Another TPT regimen using 4‐months of rifampin (RIF) was non‐inferior to the 9‐month regimen of isoniazid for the prevention of active tuberculosis in adults and was associated with higher rates of treatment completion and better safety. This study was done in a large cohort of adults, however, it contained relatively few PLHIV 39.

TPT‐related adverse events vary depending on the regimen used but they are considered manageable from a public health perspective. In the case of INH, despite occurring quite commonly, the considerable majority are low grade, transient and do not influence treatment adherence and completion 40. A recent meta‐analysis demonstrated that the three‐month isoniazid‐rifapentine regimen was associated with similar risk of adverse events and treatment discontinuation than other latent tuberculosis infection regimens 41. Regarding drug‐drug interactions (DDI) between TPT regimes and the existing ART, there are still some knowledge gaps to cover, such as co‐administration of TAF with any rifamycin, or use of 1HP with DTG. Rifampicin is a potent inducer of cytochrome P450 oxidase system, which reduces concentrations of many non‐nucleoside reverse transcriptase inhibitors (NNRTI), such as nevirapine, all protease inhibitors (PIs), and integrase inhibitors. Co‐administration with tenofovir alafenamide (TAF) decreases plasma exposure of TAF, but a healthy volunteer study with 23 participants demonstrated that intracellular levels are actually higher with TAF plus rifampicin compared to tenofovir disoproxil fumarate without rifampicin 42. Further study of TAF with rifampicin in patients with HIV infection is planned. In adults, doubling the dose of dolutegravir (50 mg twice daily rather than 50 mg once daily) with rifampicin containing TB treatment achieves adequate drug concentrations and it is likely that the same is true in older children, in whom drug disposition is similar to adults (Table 1).

**Table 1:** Tuberculosis preventive therapy and antiretroviral therapy co‐administration

TPT regimen	WHO Recommendation for the TPT regimen	Recommended for children	Compatible ART	Supporting evidence and ongoing DDI trials	Knowledge gaps
IPT	Strong recommendation	Any age	Any		Ideal length of treatment Use in pregnancy
3HR	Strong recommendation	Any age	Any NRTI, possibly including TAF EFV 600 mg QD EFV 400 mg QD with HR DTG 50 mg BID (Adults only) RAL 800 BID	RIFT (TAF + RIF in HV) study 42 STRIDE study 43 Cerrone et al. 37 INSPIRING trial ongoing 44 Taburet et al.^45^	TAF/FTC + RIF in patients with HIV – study in progress in South Africa
3HP	Conditional recommendation	>2 years old only	EFV 600 mg QD DTG 50 mg QD RAL 400 mg BID	Farenc et al. 46 DOLPHIN study 36 Weiner et al. 47	3HP dosing for children <2 years old 3HP with TAF – healthy volunteer study in progress at US NIH NCT03510468
1HP	Under review	>13 years old only	EFV 600 mg QD	BRIEF‐TB PK study 48	1HP dosing for children <13 years old 1HP with TAF 1HP with dolutegravir – ACTG trial in development (A5372)

1HP, one month daily isoniazid and rifapentine; 3HP, 3 months weekly isoniazid and rifapentine; 3HR, 3 months isoniazid and rifampin; ART, antiretroviral therapy; DDI, drug‐drug interaction; DTG, dolutegravir; EFV, efavirenz; HV, healthy volunteer; IPT, isoniazid preventive therapy; NRTI, nucleoside reverse transcriptase inhibitor; PK, pharmacokinetics; RAL, raltegravir; TAF, tenofovir alafenamide; TPT, tuberculosis preventive therapy; WHO, World Health Organization.

Children and pregnant women living with HIV must get dedicated efforts to increase access to TPT anchored to HIV programmes and maternal and child services 27, 49, 50. While most evidence is based on a 6‐month INH course; in children, the use of 3‐months daily isoniazid and rifampicin (3HR) regimen with the new paediatric TB formulations offers equal effectiveness, better adherence and fewer adverse events than standard IPT 27.

HIV positive pregnant women are at especially high risk of progression from TB infection to disease 51. TB is a significant cause of infant and maternal morbidity and mortality, especially in high‐burden settings, and has been associated with increased risk of mother‐to‐child transmission of HIV 52, 53. Treatment guidelines generally recommend use of the same regimens and dosages for pregnant women and non‐pregnant adults. However, the safety, efficacy, and appropriate timing of TPT in pregnant women living with HIV remain uncertain 54. One of the few PK studies conducted in pregnant women showed only modest changes with rifampin, so dose adjustment is not needed 55. A randomized trial of IPT during or after pregnancy in 956 HIV‐infected women on ART showed higher than expected rates of treatment‐related adverse events, and excess adverse pregnancy outcomes in the immediate IPT arm 54. However, in a post‐hoc analysis of 3HP in 112 pregnant women, the rates of spontaneous abortion and birth defects were similar to those in the general US population 56.

The knowledge gap persists in children under the age of three years: with the use of LPV/r in first line regimens (efavirenz cannot be given), options are very limited for rifamycin‐based TPT 57. A dosing recommendation for dolutegravir with rifampicin cannot yet be made.

The question of the need for repeated courses of TPT in PLHIV is not yet answered, but investigation is going in a randomized, controlled trial of two annual courses of 3HP compared to a single course, or to six months of INH in more than 4000 participants 58. There is likewise limited evidence on the best TPT regimens for drug‐resistant TB (DR‐TB), WHO provides a conditional recommendation and studies are underway 27, 59, 60, 61. Recent modelling has estimated that three in every 1000 people globally carry latent multidrug‐resistant tuberculosis infection, prevalence is around ten times higher among those younger than 15 years and there is inadequate understanding of DR‐TB infection in the context of HIV. Treatment options for multidrug‐resistant latent tuberculosis infection will be central to contribute to TB elimination 62.

Future TPT options include long‐acting drug formulations, with the potential benefit of extended duration for drug delivery that could lead to improved adherence and outcomes 63. Precedents come from the HIV therapy field and the development of an injectable combination of rilpivirine/cabotegravir that can be administered every one to two months, and ongoing studies of other injectable formulations such as cabotegravir for pre‐exposure prophylaxis 63, 64, 65, 66, 67. Since 2015, a working group on long acting/extended release formulations for TB prevention and treatment was established under the umbrella of the Long‐Acting/Extended Release Antiretroviral Resource Programme (LEAP) 68. The group has developed the target product profile for TPT formulations and is contributing to the efforts of building the pipeline for long‐acting formulations using new and existing TB drugs.

### 2.2 Programmatic opportunities to scale‐up TPT in HIV programmes

Successful TB/HIV control strategies in high‐burden settings pivot around strong collaborations and ownership between HIV and TB stakeholders 23, 69.

Programme managers, clinicians and community workers in integrated TB/HIV programmes have to be equipped and supported to become the real foundation for a robust TPT programmatic rollout 23. While increasing training and on‐the‐job mentorship, programmes could use routine data, triggering quality cycles, to gather in‐depth understanding of the successes and gaps along the cascades of care. Tailoring support to recipients of TPT will assure better adherence and management of events arising during the course of the treatment. Tracking and reporting TPT outcomes is critical, not only at individual level, but also programmatically. The quality of TPT programmes should be evaluated by their capacity to attain the highest completion rates, provide adequate clinical and programmatic monitoring and decrease causes of non‐completion, such as death, development of active TB, adverse effects or loss to care. From a global perspective, countries with high TB/HIV burden must include a minimum set of indicators for TPT programmes to their routine reports to WHO and UNAIDS, as a strategy to assure progress and accountability to the UNHLM targets.

The use of optimized, simplified and integrated diagnostic algorithms identify two pathways: PLHIV who screen TB positive will need to complete TB disease diagnostic confirmation steps, and, if positive, be initiated anti‐TB therapy. The majority of PLHIV living in high burden areas, where TB screening is negative, should be offered a TPT course. Improvements must assure sustainability and efficient use of diagnostic platforms such as Xpert and urine TB‐LAM 70 or adaptation of clinical protocols to minimize concerns about unmasked TB after ART initiation 70, 71, 72.

TPT regimens can be optimized to decrease pill burden, become shorter, patient‐friendly and decrease side effects as means to maximize treatment completion rates 22. New TPT options will be a game changer, facilitating programmatic rollout and improving engagement in care until completion. Despite the existing operational challenges related to availability of rifapentine, global stakeholders and Ministries of Health are committing to scale‐up shorter TPT regimens, making generic formulations available and reducing prices 8. National supply chains should ensure that diagnostics and treatment options for TB and HIV are available without interruption.

### 2.3 Field perspectives to scale‐up TPT in HIV programmes: differentiating care and demand creation

HIV programmes can enable environments that enhance TB integrated services at every possible opportunity as PLHIV engage or re‐engage in chronic care. Traditional models of care are expanding approaches that include DSD, creating client‐centred approaches that simplify and adapt services across the cascade of care in ways that both serve the needs of PLHIV, reduce burden on the health system and enable reallocation of resources to reach even more people 73, 74. These models vary the location of the service (e.g. facility‐based, community), reduce the frequency of encounter (e.g. 2‐monthly to 6‐monthly), and distributes tasks among doctors, nurses, pharmacists, lay healthcare workers and peers 75. Differentiation of HIV testing or ART delivery is being widely used today to improve services and to respond to the needs of specific populations for thousands of stable PLHIV in countries such as Mozambique, South Africa, Malawi or Ghana 74, 76, 77, 78, 79, 80, 81. In the era of differentiated care, programmatic scale up of TPT can be supported with the creation of integrated care models that incorporate TB screening and provision of TPT without increasing the frequency of clinic attendance, reflecting the balance for public health approaches and people's needs that are contextual and change over time 82. Applying differentiated frameworks in HIV programmes to rollout TPT can promote the creation of context‐adapted solutions to the “how” TPT is delivered within chronic ART care, “who” does TB screening and delivers TPT, “when” is TB screening done and TPT delivered and “what” package of interventions is included to achieve best TPT completion rates (Table 2).

**Table 2:** Differentiated service delivery applied to the integration of TPT in HIV programmes

	What	Where	When	Who	Target population
TB screening	Clinical screening package	At facilities Active case finding in the communities In differentiated care models of ART delivery	At every consultation with a clinician At every encounter with a community health worker	Community‐based or facility‐based health workers Peers Expert clients	PLHIV
Initiation TPT	Identify preventive regimen	Health facilities and community settings	After negative TB screening	Nurses, clinicians	PLHIV with negative TB screening and no contraindications
Follow‐up	Clinical monitoring Reporting/management of side effects Reporting of treatment completion Programmatic quality improvement	Clinical consultation, peer support Task shifted and simplified models of care	During TPT course as per agreed protocol	Nurses Expert clients Peers Community health workers	PLHIV on TPT

Increasing the demand for TPT in PLHIV will also support the path to achieve the very ambitious global TPT targets through a robust programmatic scale‐up; in many settings this will include setting up strong collaborations with the private sector or other providers of care 83. Experience with other prevention programmes such as population‐wide tobacco control or preventive voluntary male circumcision shows that social and behaviour change can be modulated with the use of ambitious communications strategies that identify and address the demand‐side barriers, increasing awareness and aligning demand with service delivery 83, 84, 85.

The lower rifapentine price recently established by the agreement between Sanofi, Unitaid, and the Global Fund, combined with initiatives such as IMPAACT4TB will contribute to catalyse change and improve uptake by facilitating access and generating evidence for programmatic use 86, 87. Advocacy (e.g. with community leaders, TB champions, healthcare workers), communications through different channels (e.g. television, radio, print media, interpersonal communication, road shows, social media, SMS reminders, household visits), and community engagement and mobilization will also be essential tools 85. Moreover health workers are in a unique position to refer and initiate TPT in PLHIV 84. Once mobilized, extensive existing networks of community‐based and health facility‐based professionals in each setting can contribute to increase awareness, remove major health system barriers and support PLHIV to start and complete TPT. As there is no “one size fits all,” qualitative research and baseline situational analyses can assist identifying context‐tailored strategies and unveil breakthroughs in different settings.

## 3 Conclusions

Revitalized approaches to TB prevention that significantly expand the range of TPT options, combined with the integration in people‐centred models of care and a solid engagement with communities, will contribute to decrease morbidity and mortality in HIV‐associated TB. HIV programmes are at an important crossroad to continue improving HIV quality of care while adding a meaningful contribution to the global TB elimination efforts.

## Competing interests

LGF, EC, VS, GB, EG, WV, SA, SS1, APL, BG, have no competing interests. GJ participated in the WHIP3TB trial evaluating 3HP given once or annually and the DOLPHIN trial that evaluated the safety and PK of 3HP given with dolutegravir in people with HIV. Sanofi donated isoniazid and rifapentine for these trials. GJ also attended a Sanofi Advisory Board on rifapentine without any compensation. AK currently manages grants from Sanofi. SS2 reports research grants to her institution from ViiV Healthcare.

## Authors' contributions

LGF and EC drafted the first draft of the manuscript, developed the concepts with input from co‐authors and managed revisions. VS, GJ, GB, EG, WV, SA, SS1, AK, APL, BG and SS2 discussed key ideas and concepts forming the basis of this commentary. SS2 developed Table 1. All co‐authors reviewed and gave input on the final draft, and cleared it for submission.
