# ERRATUM

**DOI:** 10.1002/jia2.25464

**Published:** 2020-02-20

In the article 1, the following errors were published in the text on e25438.

1. In the Introduction, the renewed targets to provide TB preventive therapy (TPT) to 30 million people should be by 2022 instead of 2020.

The sentence reads:

In 2018, the UN High‐Level Meeting (UNHLM) on Tuberculosis galvanized the global efforts to prevent TB by setting renewed targets to provide TB preventive therapy (TPT) to 30 million people by 2020 including six million PLHIV [6,7].

The sentence should have read:

In 2018, the UN High‐Level Meeting (UNHLM) on Tuberculosis galvanized the global efforts to prevent TB by setting renewed targets to provide TB preventive therapy (TPT) to 30 million people by 2022 including six million PLHIV [6,7].

- There were minor typesetting errors throughout the article and have been corrected in the online version of the article.
